# Protocol for phospho-SrcKD: rPTPεD1 complex preparation and BLI binding assays to demonstrate their exosite interface

**DOI:** 10.1016/j.xpro.2024.103046

**Published:** 2024-07-02

**Authors:** Nadendla EswarKumar, Kumar TewarySunil, Meng-Chiao Ho

**Affiliations:** 1 Institute of Biological Chemistry, Academia Sinica, 128 Academia Road Sec. 2, Nankang, Taipei 115, Taiwan; 2 Department of Immunology, St. Jude Children’s Research Hospital, Memphis, TN 38105, USA; 3 Institute of Biochemical Sciences, National Taiwan University, Taipei 106, Taiwan

**Keywords:** signal Transduction, protein expression and purification, structural Biology

## Abstract

Here, we present a protocol for the *in vitro* phosphorylation of Src kinase domain (SrcKD), preparation of phospho-SrcKD in complex with the D1 domain of rPTP epsilon (rPTPεD1), and binding assays using biolayer interferometry (BLI). We describe steps for the *in vitro* phosphorylation of SrcKD and preparation of the phospho-SrcKD: rPTPεD1 complex for small-angle X-ray scattering (SAXS) experiments. We then detail instructions for the BLI binding assay to determine the binding affinity between phospho-SrcKD and rPTPεD1.

For complete details on the use and execution of this protocol, please refer to EswarKumar et al.^1^

## Highlights

• Detailed protocol for SrcKD *in vitro* phosphorylation
• Detailed protocol for phospho-SrcKD: rPTPεD1 complex preparation
• Detailed protocol for phospho-SrcKD: rPTPεD1 binding assays

## Abstract (editor highlights)

Publisher’s note: Undertaking any experimental protocol requires adherence to local institutional guidelines for laboratory safety and ethics.

## Graphical abstract

**Figure:**
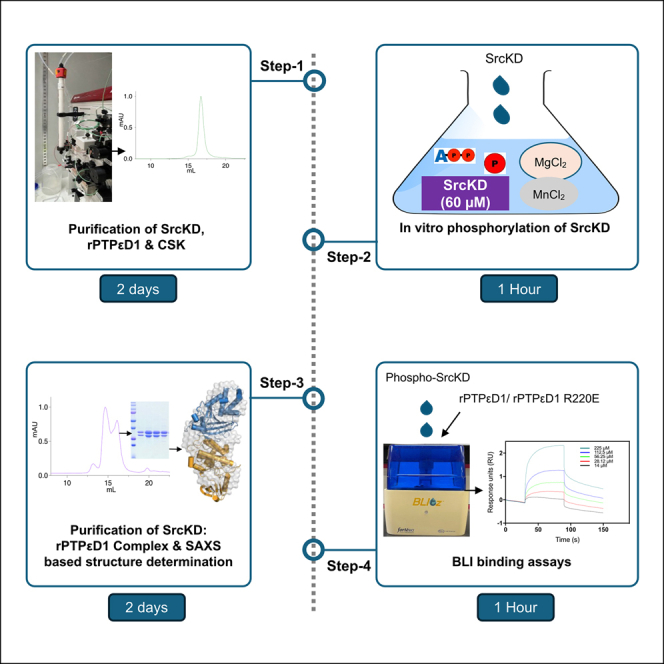

## Before you begin

To analyze the function and dynamics of phospho-protein: protein complexes, it is crucial to understand their structures.^2^ However, the phospho-protein: protein interactions involved in the signaling pathway make this difficult as they are highly transient and prone to easy dissociation,^3^ and their complex preparation at the milligram scale for structure determination is challenging. The current protocols detail the steps needed to purify the phospho-SrcKD: rPTPεD1 complex and perform BLI binding assays to validate the identified interface. They follow those published in EswarKumar et al.^1^^,^^4^ In outline, Src-KD, rPTPεD1 & C-terminal Src kinase (CSK) genes are cloned and expressed in a bacteria expression system. The proteins are then purified using immobilized metal affinity and size exclusion chromatography (please refer to EswarKumar et al. for a detailed protocol^1^). First, the phosphorylated form of SrcKD is prepared, which allows us to purify the phospho-SrcKD: rPTPεD1 complex by size-exclusion chromatography for SAXS experiments. For the binding assays, phosphorylated SrcKD and rPTPεD1 are purified separately, and the association rates are determined using BLI.

## Key resources table

**Table:** 

REAGENT or RESOURCE	SOURCE	IDENTIFIER
**Chemicals, peptides, and recombinant proteins**		

cOmplete protease inhibitor cocktail tablets	Roche	REF# 04693132001
Superdex 200 increase 10/300 column	GE Healthcare	LOT# 290117342-AC
Superdex 75 increase 10/300 column	GE Healthcare	Lot# 10244369
Ni-NTA agarose	QIAGEN	Lot# 172030479
Amicon ultra centrifugal filter (MWCO, 10 kDa)	Merk Millipore Sigma	R1EB08895
ATP	Acros Organics	CAS# 34369-07-8
LB medium	Difco	LOT# 2213695
Ni-NTA biosensor tips (ForteBio Inc.)	ForteBio (Pall Life Sciences)	N/A
DTT	Cyrusbioscience	LOT# 305111
Chitin resin	New England Biolabs	Catalog# S6651S
M6-6His-Src kinase domain (Trp 260-Leu 533) - K295M/Y416F	EswarKumar et al.^1^	N/A
pET9a-His-MBP-rPTPεD1 domain (Ser 101-Thr 400)-C335A	EswarKumar et al.^1^	N/A
pET9a-His-MBP-rPTPεD1 domain (Ser 101-Thr 400)-C335A/R220E	EswarKumar et al.^1^	N/A
pET9a-His-MBP-rPTPεD1 domain (Ser 101-Thr 400)-C335A/K237D	EswarKumar et al.^1^	N/A
M6-6His-Src kinase domain (Trp 260-Leu 533) - K295M/Y416F/E486A	EswarKumar et al.^1^	N/A
M6-6His-Src kinase domain (Trp 260-Leu 533) - K295M/Y416F/K518D	EswarKumar et al.^1^	N/A
pET-Duet-CSK-His-CBD	EswarKumar et al.^1^	N/A

**Bacterial and virus strains**		

*Escherichia Coli* BL21	Thermo Fisher Scientific	Cat# C600003

**Software and algorithms**		

GraphPad Prism7	Hayes, Laue et al. 1995, Schuck 2000	N/A
BLItz Pro software	ForteBio (Pall Life Sciences)	N/A

**Other**		

Beckman Avanti J25	Beckman Coulter	JA25.50 rotor
AKTA purifier	GE healthcare	N/A
BLItz	ForteBio	N/A

## Materials and equipment

**Table:** Ni-NTA lysis buffer

Reagent	Final concentration	Amount
Phosphate buffer pH 7.5	100 mM	N/A
NaCl	500 mM	N/A
Glycerol	10%	N/A
β-Mercaptoethanol	10 mM	N/A
Protease inhibitor cocktail	1 tablet per 50 mL solution	N/A
**Total**	N/A	N/A

Lysis buffer should not be stored. Use a freshly prepared.

**Table:** Ni-NTA elution buffer

Reagent	Final concentration	Amount
Tris-HCl pH 7.5	50 mM	N/A
NaCl	500 mM	N/A
Imidazole	500 mM	N/A
**Total**	N/A	N/A

Store buffer at 4°. Maximum storage time of one month. Check for contaminant growth prior to use.

**Table:** SEC buffer

Reagent	Final concentration	Amount
Tris-HCl pH 7.5	20 mM	N/A
NaCl	150 mM	N/A
DTT	5 mM	N/A
**Total**	N/A	N/A

SEC buffer should not be stored. Use a freshly prepared and filtered SEC buffer.

**Table:** BLI kinetics buffer

Reagent	Final concentration	Amount
Tris-HCl pH 7.5	20 mM	N/A
NaCl	50 mM	N/A
DTT	5 mM	N/A
**Total**	N/A	N/A

BLI kinetic buffer should not be stored. Use a freshly prepared.

## Step-by-step method details

### *In vitro* phosphorylation of SrcKD

**Timing: 2 h**

**Figure 1:**
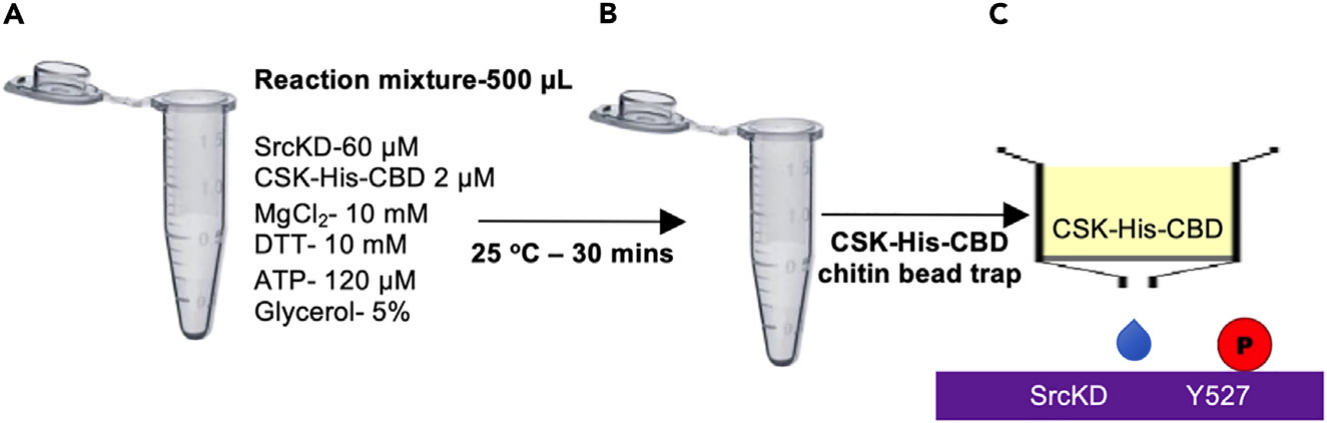
Protocol for *in vitro* phosphorylation SrcKD (A) Step 1: Preparation of reaction mixture for *in vitro* SrcKD phosphorylation. (B) Step 2: Incubation of the reaction mixture at room temperature for 30 min. (C) Step 3: Separation of phospho-SrcKD from the mixture using chitin beads trap.

To determine the structure of the phospho-SrcKD: rPTPεD1 complex and to demonstrate the mechanism of their interaction, phospho-SrcKD and rPTPεD1 are produced separately. It is already established that CSK phosphorylates SrcKD.^5^ This section describes SrcKD phosphorylation by CSK in the presence of ATP, MnCl_2_, and MgCl_2_ (Figure 1).

1. First, clone SrcKD with an N-terminal His tag (His-SrcKD), and CSK with a His tag and a chitin-binding domain (CBD) at the C-terminus (CSK-His-CBD).
2. Use immobilized metal affinity chromatography (IMAC) and size-exclusion chromatography (SEC) to purify both His-SrcKD and CSK-His-CBD.
  a. Collect the pure fractions containing His-SrcKD, pool them together, and treat with His-TEV protease (1 mg of TEV per 50 mg of fusion protein) to remove the His tag.^6^
  b. Purify the resulting tag-free SrcKD, digested His-tag, and TEV protease proteins further with a second IMAC.
  c. Concentrate the flow-through from the second IMAC containing SrcKD and further purify using the HiLoad Superdex 200 16/600 SEC column.

***Note:*** Before use, the column should be pre-equilibrated with SEC buffer (All the buffers used in this protocol are listed in Table 1).

3. Concentrate the purified SrcKD and CSK-His-CBD to 60 μM and 2 μM, respectively for phosphorylation.
4. To achieve the phosphorylation of SrcKD, add 10 mM MgCl_2_, 10 mM MnCl_2_, 10 mM DTT, 120 μM ATP, 2 μM CSK-His-CBD and 5% glycerol in 1 mL of SrcKD solution with a final concentration of 58 μM.
5. Incubate the reaction mixture at 25°C for 30 min.
6. Next, use chitin beads to get rid of the CSK-His-CBD from the reaction mixture containing phosphorylated SrcKD and CSK-His-CBD.

***Note:*** Pre-equilibrated the chitin beads with 20 mM Tris pH 8.0, 50 mM NaCl, 10 mM MgCl_2_ and 5 mM DTT

7. Now, the flow-through contains phosphorylated SrcKD for complex formation with rPTPεD1.

***Note:*** At least 5 mM DTT needs to be added during the process. We observed SrcKD precipitation with less than 5 mM DTT.

**Table 1:** Affinities (K_D_) & association/dissociation parameters (*k*_on/off_) of the wild type & variants

Name of the protein	K_on_ (1/Ms)	K_off_ (1/s)	K_D_ (M)
**rPTPεD1 wild type**	1.083E+3	2.838E-2	2.62E-5
**rPTPεD1-R220E**	2.024E+2	3.409E-2	1.68E-4
**rPTPεD1-K237D**	2.423E+2	8.534E-2	3.522E-4
**SrcKD-E486A**	5.922E+2	4.758-2	8.036E-5
**SrcKD-D518K**	3.598E+2	6.613E-2	1.838E-4

### Purification of the phospho-SrcKD: rPTPεD1 complex

**Timing: 2 h**

**Figure 2:**
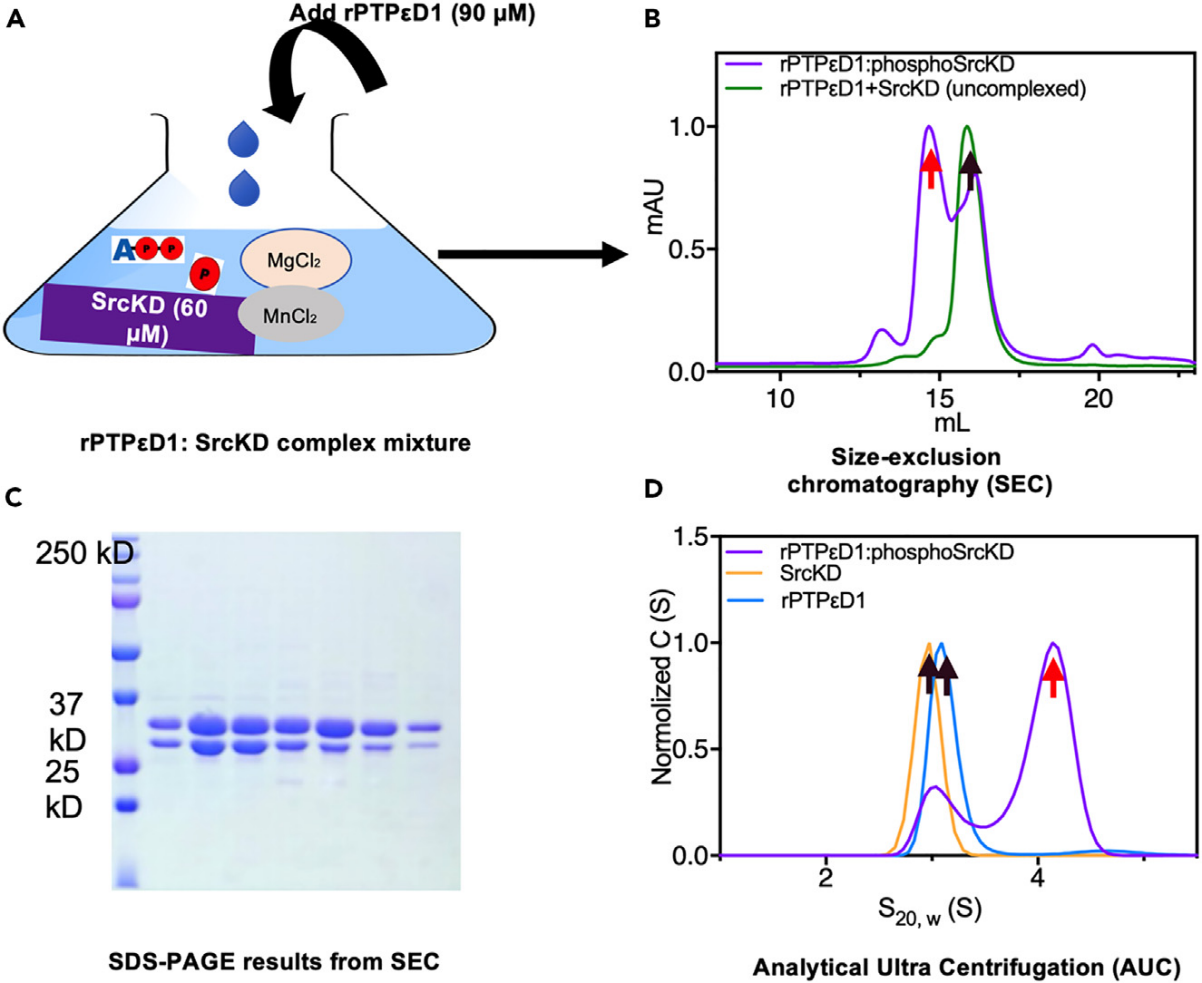
Purification of phospho-SrcKD:rPTPεD1 complex (A) Illustration of *in vitro* phosphorylation of SrcKD and preparation of the phosphor-SrcKD: rPTPεD1 complex. (B) An overlaid SEC profile of the phospho-SrcKD: rPTPεD1 complex colored in violet and a mixture of rPTPεD1 and SrcKD (un-complex form) colored in green. The elution positions of complex and un-complexed proteins are indicated as red and black arrows, respectively. The SEC was performed using the Superdex 200 10/300 GL column. (C) The SDS-PAGE result from the SEC of the phospho-SrcKD: rPTPεD1 complex. The first lane is the reference marker with the corresponding MW shown. Lanes 2–4 correspond to the highest peak area and lanes 5–8 correspond to the second peak area from the SEC (D) Distribution of the sedimentation coefficients of the rPTPεD1, phospho-SrcKD, and phospho-SrcKD: rPTPεD1 complex are shown in blue, yellow and violet, respectively. The complex revealed an additional peak with a S_20,w_ value of 4.11.
9. Separate the phospho-SrcKD: rPTPεD1 complex from un-complexed proteins with the Superdex 200 Increase 10/300 column at 4°C in the presence of SEC buffer using the AKTA purifier.

***Note:*** Pre-equilibrate the column with SEC buffer prior to the experiment.

8. Mix the phosphorylated SrcKD (phospho-SrcKD) with 0.5 M excess of purified rPTPεD1 and incubate for 10–30 min at 25°C (Figure 2A).

10. Inject 500 μL of sample volume into the AKTA purifier at a flow rate of 0.5 mL per minute.
11. Confirm the phospho-SrcKD: rPTPεD1 complex formation by elution volume, SDS-PAGE electrophoresis, and analytical ultra-centrifugation (AUC).
  a. The molecular weights of SrcKD and rPTPεD1 are similar, so the un-complexed form of unphosphorylated SrcKD and rPTPεD1 elutes at 15.6 mL, whereas the phospho-SrcKD: rPTPεD1 complex elutes at 14.7 mL.
  b. Almost a 1 mL peak shift between the complex and individual proteins in SEC is required based on their molecular weights, as shown in Figure 2B.
  c. SDS-PAGE results show two corresponding bands representing the rPTPεD1: phospho-SrcKD complex (Figure 2C).
  d. The AUC results should be consistent with SEC results. The individual SrcKD and rPTPεD1 show a single peak with an S_20_ of ∼ 3, whereas the mixture of phospho-SrcKD: rPTPεD1 shows an additional peak with S_20_ value > 4 (Figure 2D).

***Note:*** Using no more than 50 mM NaCl in the buffer is crucial to the preservation of the rPTPεD1: SrcKD complex. The presence of 100 or 150 mM NaCl in the buffer causes the complex to dissociate.

### The binding assays by biolayer interferometry

**Timing: 4 h**

12. To perform the binding experiments, the rPTPεD1, phospho-His-SrcKD, and the variants (Table 1) need to be purified.

***Note:*** The rPTPεD1 and its variants are untagged. The protein variants are listed in the key resources table.

**Figure 3:**
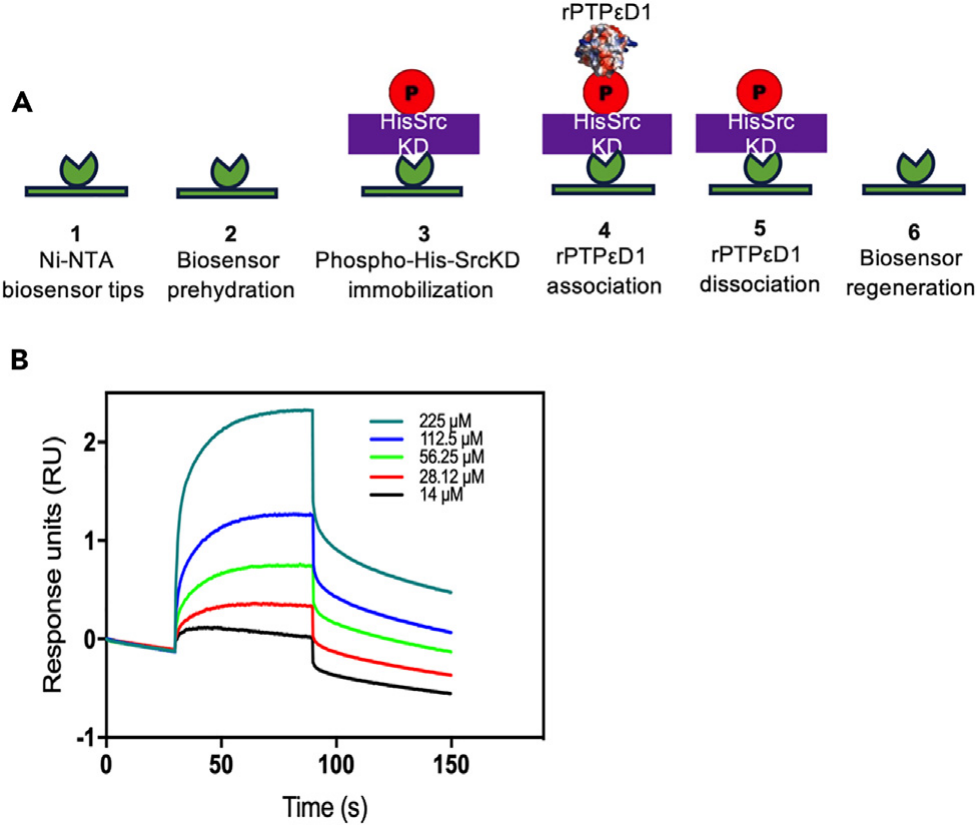
Illustration of the binding affinity between phosphor-His-SrcKD and rPTPεD1 by BLITz (A) Illustration of the detailed BLI binding protocol. (B) Example Blitz sensorgrams of various concentrations of rPTPεD1 binding to immobilized phospho-His-SrcKD.
15. First, pre-hydrate the Ni-NTA sensors for 10 min in the BLI kinetics buffer.
16. After hydration, attach the Ni-NTA biosensor to the BLI system and initialize baseline measurements for 30 s.
17. Load 5 μL of the phospho-His-SrcKD (at a concentration of 58 μM) as bait into the BLI drop holder and immobilize for 3 min on the Ni-NTA sensor tips.
18. Wash the immobilized phospho-His-SrcKD with 250 μL of the BLI kinetics buffer loaded into the system before the BLI signal was measured for 90 s to prepare the biosensor for interaction with its partner.

***Note:*** To maintain a stable phosphorylation level of His-SrcKD, supplement the buffer with a fresh 100 μM ATP and 1 μM CSK before every immobilization.

13. The Ni-NTA biosensor tips on the BLItz system by ForteBio Inc. are used to measure the binding kinetics between rPTPεD1 and phospho-His-SrcKD.
14. The experiments can be divided into four stages:
  a. Baseline measurements with BLI kinetic buffer (steps 1, 2),
  b. Loading phospho-His-SrcKD (or variants) onto the Ni-NTA biosensor (steps 3 to 5),
  c. Association with the binding partner, rPTPεD1 (or its variants) (step 6).
  d. Dissociation of the binding partner (step 7) (Figure 3).

19. Once the bait protein reaches saturation, load 5 μL of the analyte (rPTPεD1 or its variants) into the BLI drop holder. Lower the arm to initiate association and measure the BLI signal for 120 s.
20. Later, add 250 μL of BLI kinetic buffer into the 0.5 mL microfuge tube and lower the biosensor to initiate dissociation and measure the BLI signal for 120 s.
21. After the final measurements, process the sensorgram using the BLItz Pro software (ForteBio Inc.).
22. For data analysis, select global fitting and non-linear regression analysis. Examine the fitting results by observing the overlay of regression curves on sensor data traces.

***Note:*** For reference, a table with determined parameter values such as rate constants, R_max_, K_A_, K_D_ (Table 1) and statistics like the standard error of parameters and Chi-squared is outputted.

23. Lastly, export the data to text files and generate publication-quality sensorgrams using GraphPad Prism version 7 software^7^^,^^8^

***Note:*** The hydrolysis of phosphorylation on protein is a danger that needs to be avoided. Maintaining phosphorylation is critical to obtaining reliable binding constants. Fresh ATP solution and stable CSK should be supplemented before every immobilization. All steps in binding experiments should be carried out promptly and any waiting time should be avoided.

## Expected outcomes

By following this protocol, recombinant SrcKD and rPTPεD1 can be purified and phosphorylated SrcKD obtained in around 2 weeks. The phospho-SrcKD: rPTPεD1 complex at mg scale can be prepared for structural studies, and binding constants between phospho-Src and rPTPεD1 can be determined. This protocol will be useful to researchers studying phospho-protein: protein interactions.

## Limitations

Biolayer interferometry is a label-free technology to measure biomolecular interaction directly. The binding between the protein immobilized on the biosensor tip surface and the binding partner in the solution increases the optical thickness at the biosensor tip, resulting in a wavelength shift. However, any non-specific binding between the biosensor tip and the binding partner can also yield a wavelength shift. Although BLItz has a relatively short machine dead time and requires as little as 4 μL of the analyte, it is a less sensitive and low throughput instrument.

## Troubleshooting

### Problem 1

[Step 8] During complex preparation, the phospho-SrcKD: rPTPεD1 complex dissociates due to its transient nature in the presence of 100 mM or more of NaCl.

### Potential solution

Various NaCl concentrations from 50‒150 mM can be screened. Our results show that the phospho-SrcKD: rPTPεD1 complex remains stable at 50 mM NaCl, enabling its purification through SEC experiments.

### Problem 2

[Step 12] Different instruments, such as Surface Plasmon Resonance (SPR), isothermal calorimetry (ITC), and microscale thermophoresis (MST) are used to measure the binding affinities between the phospho-SrcKD and rPTPεD1 but fail to obtain accurate binding constants due to phospho-tyrosine hydrolysis on phospho-SrcKD during the long instrument dead time unavoidable with their use.

### Potential solution

Ultimately, the BLItz system with a short dead time may need to be used. In addition, ATP and CSK can be supplemented before every binding measurement, which overcame the problem in our lab.

## Resource availability

### Lead contact

Further information and reasonable requests for reagents may be directed to, and will be fulfilled by the lead contact, Meng-Chiao Ho (joeho@gate.sinica.edu.tw).

### Technical contact

Questions about the technical specifics of this protocol should be directed to the technical contact, Meng-Chiao Ho (joeho@gate.sinica.edu.tw).

### Materials availability

All unique reagents generated in this study are available upon reasonable request from the lead contact.

### Data and code availability

The data presented in the figures were generated as part of the ref. 1. This protocol does not report the original code.
